# Analysis of bacterial survival after exposure to reactive oxygen species or antibiotics

**DOI:** 10.1016/j.dib.2016.03.060

**Published:** 2016-03-22

**Authors:** Joris van der Heijden, Stefanie L. Vogt, Lisa A. Reynolds, Jorge Peña-Díaz, Audrey Tupin, Laurent Aussel, B. Brett Finlay

**Affiliations:** a Michael Smith Laboratories, University of British Columbia, Vancouver, British Columbia, Canada V6T 1Z4; b Department of Microbiology and Immunology, University of British Columbia, Vancouver, British Columbia, Canada; c Department of Biochemistry and Molecular Biology, University of British Columbia, Vancouver, British Columbia, Canada; d Laboratoire de Chimie Bactérienne, CNRS, Institut de Microbiologie de la Méditerranée, Marseille, France

**Keywords:** Oxidative stress, Reactive oxygen species, Hydrogen peroxide, Bacterial survival, Antibiotics

## Abstract

The redox balance in a variety of Gram-negative bacteria was explored using redox sensitive GFP (roGFP2), J. van der Heijden et al. doi:10.1016/j.freeradbiomed.2015.11.029[1]. This data article provides Supporting material to further investigate the relationship between *Salmonella typhimurium* survival and oxidative stress. The first set of data presented in this article, shows the percentage of surviving bacteria after exposure to hydrogen peroxide. The second set of data shows the concentration of hydrogen peroxide that was produced by *S.* Typhimurium in different growth phases. The last set of data shows the percentage of surviving *S.* Typhimurium bacteria after exposure to different antibiotics.

## Specifications Table

**Table:** 

Subject area	*Biology*
More specific subject area	*Redox biology and microbiology*
Type of data	*Graphs*
How data was acquired	*Fluorescent plate reader (Tecan Trading AG, model: Infinite M200) to measure fluorescence for the Amplex red assay and colony forming units to assess bacterial survival*
Data format	*Analyzed*
Experimental factors	*Salmonella typhimurium (expressing roGFP2) was grown under various conditions to late exponential phase or stationary phase prior to the experiments.*
Experimental features	*The survival of Salmonella typhimurium after exposure to hydrogen peroxide was analyzed under a variety of different conditions by determining colony forming units.*
*Secondly, endogenous production of hydrogen peroxide was measured using the Amplex red assay.*
*Finally, the percentage survival of Salmonella typhimurium was determined after exposure to several antibiotics by colony forming units.*
Data source location	*Vancouver, Canada, GPS 49.2611° N, 123.2531° W*
Data accessibility	*Data is with this article*

## Value of the data

• The data identifies environmental conditions that affect bacterial survival after exposure to oxidative stress.
• The growth phase of bacteria was found to affect production of hydrogen peroxide by bacteria.
• The data provides an analysis of bacterial survival after exposure to different antibiotics.

## 1. Data

After growing *Salmonella* Typhimurium bacteria under a variety of environmental conditions, bacterial survival was determined after exposure to oxidative stress. Secondly, the production of endogenous hydrogen peroxide was measured of bacteria that were grown to different growth phases. Lastly, bacterial survival of *S.* Typhimurium was analyzed after exposure to different antibiotics.

## 2. Experimental design, materials and methods

### 2.1 Bacterial strain

**Fig. 1:**
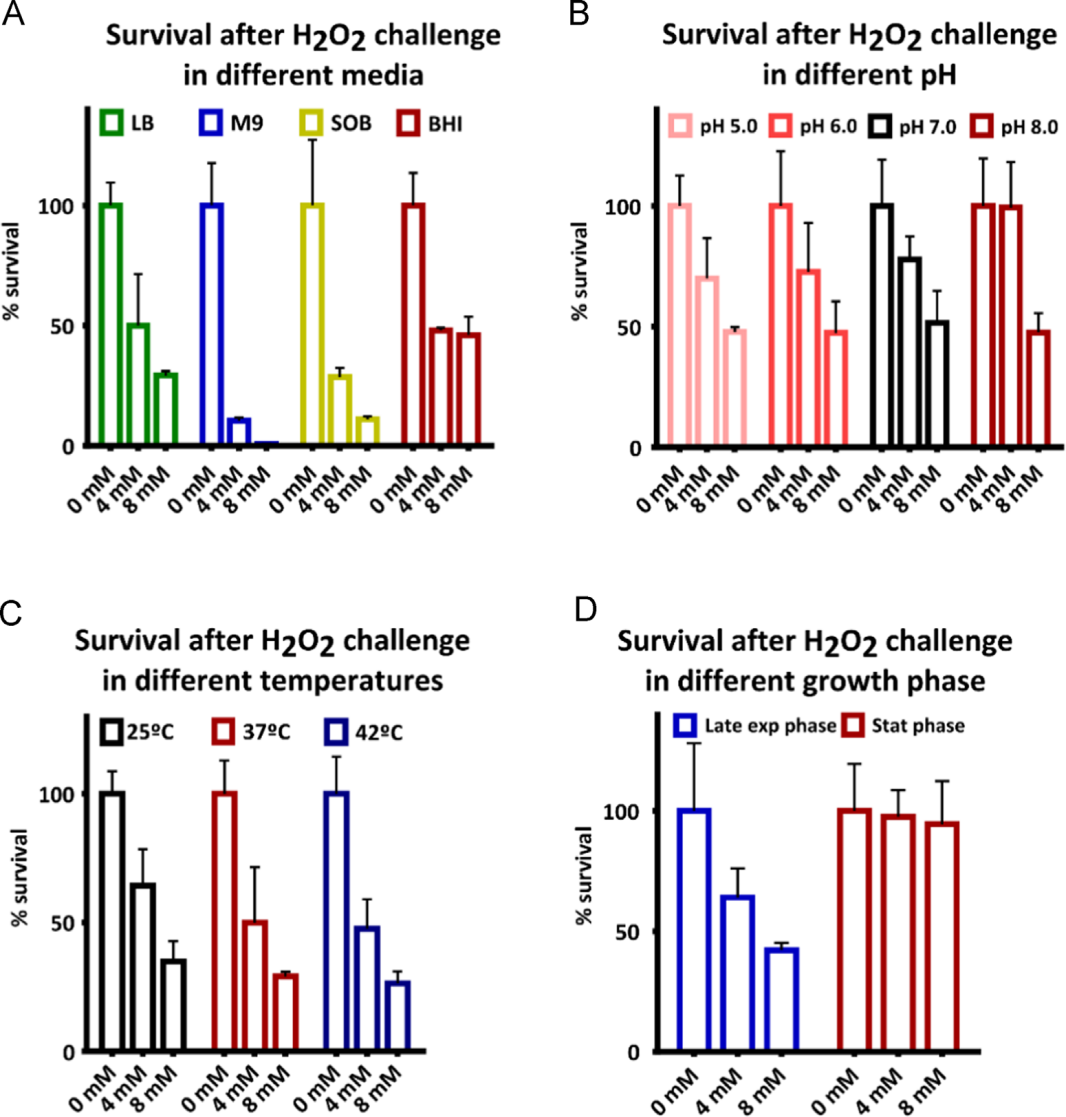
Percentage of surviving *S*. Typhimurium after challenge with 4 mM or 8 mM hydrogen peroxide. A. Bacteria were grown in four different media until reaching exponential growth phase. After growth in Luria Broth, M9 minimal medium, SOB medium or Brain and Heart Infusion medium, the bacteria were transferred to saline at OD_600_=1.0 and following a 2 h exposure to 4 mM or 8 mM of hydrogen peroxide, colony forming units were determined. Statistical significance was determined using a one-way ANOVA analysis. Each value was compared to the LB medium control. At 4 mM exposure, M9 medium and SOB medium showed significantly less survival than LB medium. At 8 mM exposure, M9 medium, SOB medium and HBI medium showed significantly less or more survival than LB medium. B. Bacteria were grown in LB until reaching exponential growth phase and transferred to phosphate buffered saline at OD_600_=1.0 of pH 5, pH 6, pH 7 or pH 8. After a 2 h exposure to 4 mM or 8 mM of hydrogen peroxide, colony forming units were determined. Statistical significance was determined using a one-way ANOVA analysis. No significant differences were observed. C. Bacteria were grown in LB until reaching exponential growth phase and transferred to saline at OD_600_=1.0. While incubating at 25 °C, 37 °C or 42 °C the bacteria were challenged with 4 mM or 8 mM of hydrogen peroxide for 2 h and after this, colony forming units were determined. Statistical significance was determined using a one-way ANOVA analysis. No significant differences were observed. D. Bacteria were grown in LB until reaching exponential growth phase or stationary phase. The bacteria were transferred into saline at OD_600_=1.0 and challenged with 4 mM or 8 mM of hydrogen peroxide for 2 h. Colony forming units were determined. In all experiments, the percentage of surviving bacteria was calculated relative to the number of bacteria without exposure to hydrogen peroxide. Each value represents the mean of six technical replicates. Statistical significance was determined using a one-way ANOVA analysis. At 4 mM and 8 mM exposure, stationary phase bacteria showed significantly increased survival compared to bacteria that were grown to exponential phase.

**Fig. 2:**
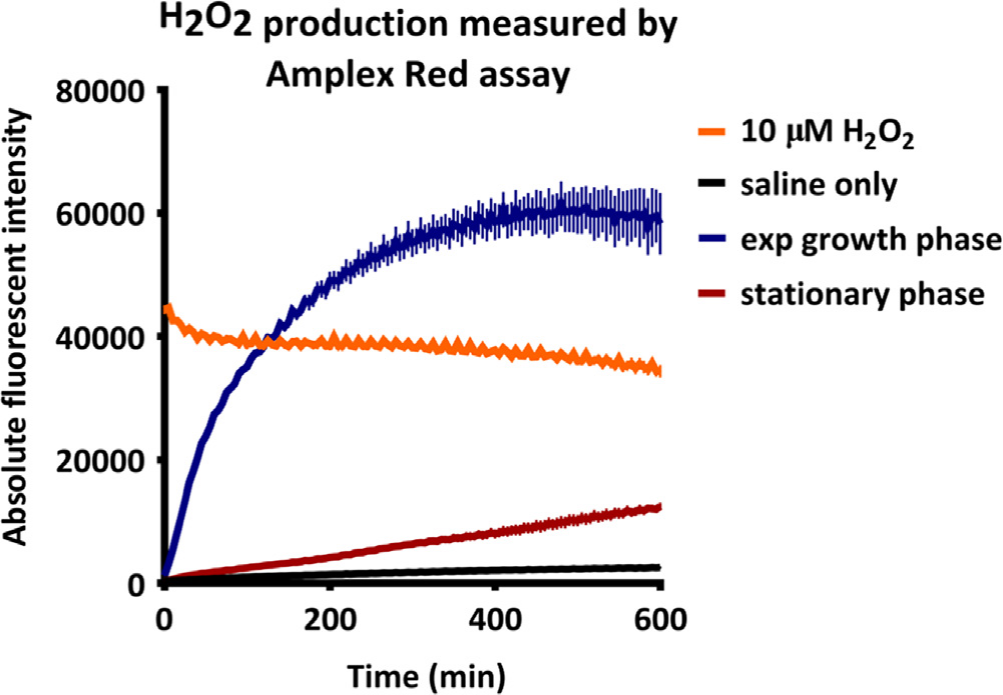
Endogenous production of hydrogen peroxide by *S.* Typhimurium in exponential growth phase and stationary phase. Prior to the experiment, *hpxF* bacteria were grown to exponential growth phase (blue) or stationary phase (red) in Luria Broth at 37 °C. Bacteria were transferred to saline at OD_600_=1.0 and endogenous production of hydrogen peroxide was measured using the Amplex red assay, by continuously measuring production in a fluorescent plate reader at 37 °C. The background levels of hydrogen peroxide were determined by measuring hydrogen peroxide in saline only conditions (black). A biologically relevant concentration of 10 μM hydrogen peroxide was included as a control (orange). Each value represents the mean of four technical replicates.

**Fig. 3:**
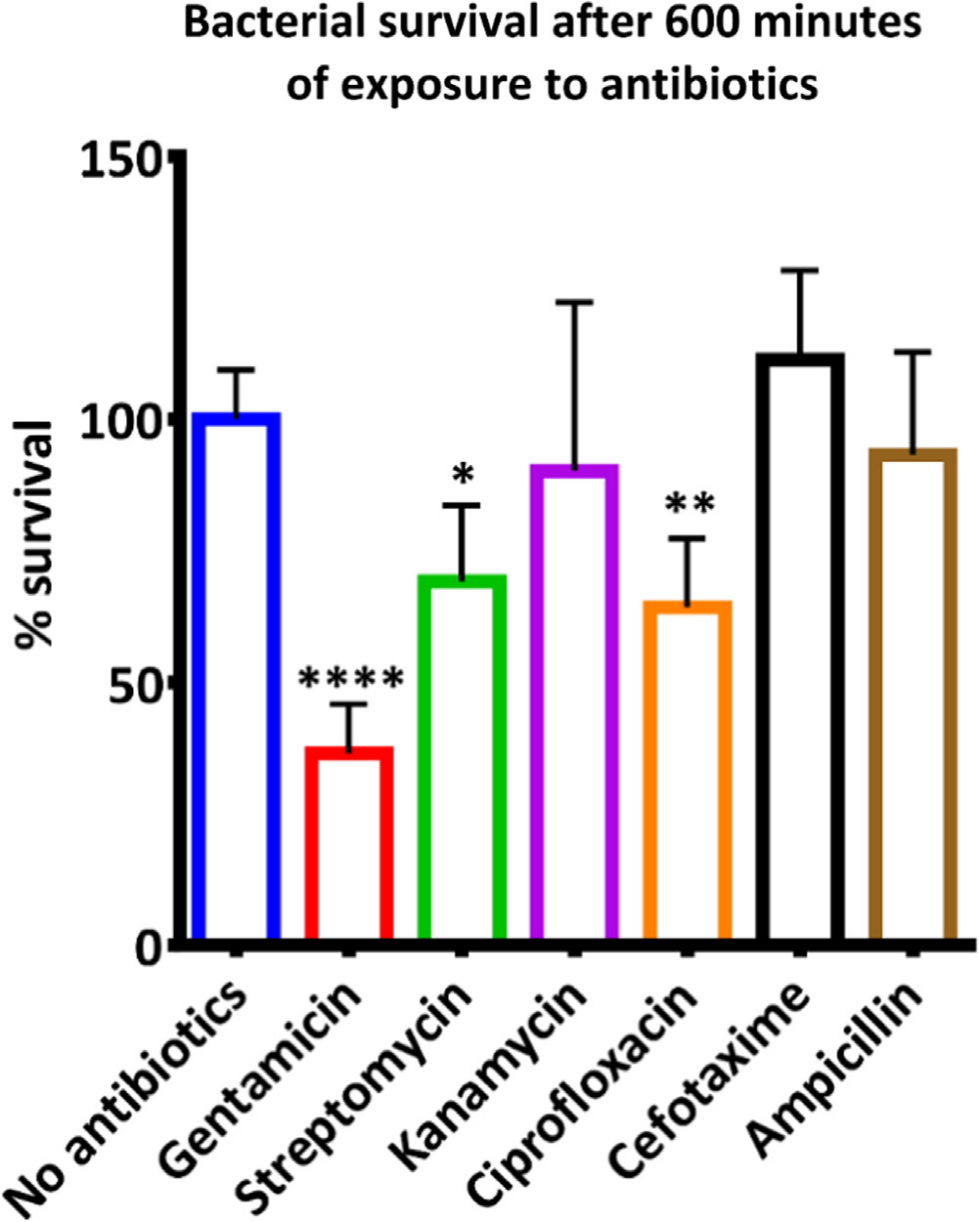
Survival of *S.* Typhimurium after exposure to a variety of antibiotics under non-growing conditions. Prior to the experiment, bacteria were grown to exponential growth phase. The colony forming units were determined after exposure to 12.5 μg/ml gentamicin, 25 μg/ml streptomycin, 25 μg/ml kanamycin, 1 μg/ml ciprofloxacin, 50 μg/ml cefotaxime, 25 μg/ml ampicillin or no antibiotics while being incubated in saline at 37 °C. The percentage survival was normalized to the survival after incubation without antibiotics. Only after exposure to gentamicin, streptomycin or ciprofloxacin, bacteria were less likely to survive under non-growing conditions. Statistical significance was determined by a one-way ANOVA analysis comparing each antibiotic to the no antibiotic control. Each value represents the mean of six technical replicates.

Experiments for Fig. 1 were performed with wildtype *S.* Typhimurium (12023). Experiments for Fig. 2, Fig. 3 were performed with *hpxf S.* Typhimurium which is devoid of catalases and peroxidases[ref 1]. During growth of *hpxf S.* Typhimurium cultures, bovine liver catalase (1000 U/ml) was added to the medium to alleviate oxidative stress as described previously [2].

### 2.2 Survival experiments after growth in different growth media

*S.* Typhimurium (12023) was grown overnight in in 5 ml LB medium. The following day, 300 μL of the overnight culture was used to inoculate 10 mL of LB medium, 10 mL of M9 medium, 10 mL of SOB medium or 10 mL of Brain Heart infusion medium while shaking at 37 °C. Bacteria were grown for 3 h in LB, SOB or Brain Heart infusion media or for 5 h in minimal M9 medium. Bacteria were then spun down and washed in saline (0.9% w/v) before resuspension of bacterial cultures at density OD_600_=2 in saline solution. 100 μL of culture was loaded into pre-warmed 96 well plate and subsequently 100 μL of saline containing 0 mM, 8 mM or 16 mM of hydrogen peroxide was added for a final concentration of 0 mM, 4 mM or 8 mM of hydrogen peroxide. Incubation with hydrogen peroxide was done in static plates at 37 °C for 2 h. After 2 h, bacteria were plated and colony forming units were determined.

### 2.3 Survival experiments under different pH

*S.* Typhimurium (12023) was grown overnight in in 5 ml LB medium. Next day, 300 μL of the overnight culture was used to inoculate in 10 mL of LB medium and bacteria were grown for 3 h to late exponential phase. After 3 h, bacteria were washed and resuspended in phosphate buffered saline at pH 5.0, pH 6.0, pH 7.0 or pH 8.0. 100 μL of culture was loaded into pre-warmed 96 well plate and subsequently 100 μL of corresponding PBS at pH 5.0, pH 6.0, pH 7.0 or pH 8.0 containing 0 mM, 8 mM or 16 mM of hydrogen peroxide was added for a final concentration of 0 mM, 4 mM or 8 mM of hydrogen peroxide. After 2 h, bacteria were plated and colony forming units were determined.

### 2.4 Survival experiments in different temperatures

*S.* Typhimurium (12023) was grown in LB as described above. 100 μL of culture in saline at density OD_600_=2 was loaded into pre-warmed 96 well plate and plates were incubated at 22 °C, 37 °C or 42 °C. After incubation for 5 minutes, 100 μL of saline containing 0 mM, 8 mM or 16 mM of hydrogen peroxide was added for a final concentration of 0 mM, 4 mM or 8 mM of hydrogen peroxide. After 2 h, bacteria were plated and colony forming units were determined.

### 2.5 Survival experiments with bacteria in exponential growth phase or stationary phase

*S.* Typhimurium (12023) was grown overnight in in 5 ml LB medium to stationary phase. The following day, 300 μL of the overnight culture was used to inoculate 10 mL of LB medium and bacteria were grown for 3 h to late exponential phase. Bacteria from the stationary phase and exponential phase cultures were washed and resuspended in saline to create bacterial cultures at density OD_600_=2.100 μL of culture was loaded into pre-warmed 96 well plate and subsequently 100 μL of saline containing 0 mM, 8 mM or 16 mM of hydrogen peroxide was added for a final concentration of 0 mM, 4 mM or 8 mM of hydrogen peroxide. After 2 h, bacteria were plated and colony forming units were determined.

### 2.6 Survival experiments after exposure to different antibiotics

*S*. Typhimurium hpxf, which is devoid of catalases and peroxidases, was grown overnight in in 5 ml LB medium supplemented with bovine liver catalase (1000 U/ml) to stationary phase. The following day, 300 μL of the overnight culture was used to inoculate 10 mL of LB medium and bacteria were grown for 3 h to late exponential phase. Bacteria were then spun down and washed in saline (0.9% w/v) before resuspension of bacterial cultures at density OD_600_=2 in saline solution. 100 μL of culture was loaded into pre-warmed 96 well plate and subsequently 100 μL of saline containing 25 μg/ml gentamicin, 50 μg/ml streptomycin, 50 μg/ml kanamycin, 2 μg/ml ciprofloxacin, 100 μg/ml cefotaxime, 50 μg/ml ampicillin or no antibiotics was added. This addition lead to a final concentration of 12.5 μg/ml gentamicin, 25 μg/ml streptomycin, 25 μg/ml kanamycin, 1 μg/ml ciprofloxacin, 50 μg/ml cefotaxime, 25 μg/ml ampicillin or no antibiotics. Cultures were incubated at 37 °C, shaking, for 10 h. After incubation, bacteria were washed in saline and plated on LB agar plates to determine colony forming units. The final numbers of colony forming units were normalized to the numbers that were obtained from the condition without antibiotics.

### 2.7 Measuring real-time production of hydrogen peroxide using the Amplex red assay

The Amplex red Hydrogen peroxide Assay (Molecular probes #A22188) was performed according to the manufacturer׳s instructions. In short, the Amplex red fluorescent dye was added to bacteria in saline that were grown to exponential phase or stationary phase and loaded on a 96 well plate. Immediately after addition of the dye, the plate was transferred to a fluorescent plate reader and fluorescence was monitored (excitation/emission maxima=570/585 nm) for 10 h at 37 °C. The background fluorescence and hydrogen peroxide levels were determined by measuring fluorescence of saline only. In order to determine if biologically relevant concentrations could be measured, a 10 μM hydrogen peroxide control was added.

## References

[1] van der Heijden J. (2015). Exploring the redox balance inside gram-negative bacteria with redox-sensitive GFP. Free Radic. Biol. Med..

[2] Hebrard M., Viala J.P., Meresse S., Barras F., Aussel L. (2009). Redundant hydrogen peroxide scavengers contribute to Salmonella virulence and oxidative stress resistance. J. Bacteriol..

